# The comparison of visibility of the maxillary sinus septa between cone-beam computed tomography scans and panoramic radiograph images as dependent on the cortical bone thickness: a retrospective comparative study

**DOI:** 10.1186/s40729-024-00542-1

**Published:** 2024-05-07

**Authors:** Ali Reza Ketabi, Stefan Hassfeld, Hans-Christoph Lauer, Andree Piwowarczyk

**Affiliations:** 1 Department of Prosthodontics, School of Dentistry, Faculty of Health, Witten/Herdecke University, Alfred-Herrhausen-Straße 45, 58455 Witten, Germany; 2 Private Dental Office of Dr Ali-Reza Ketabi, Epplestraße 29 a, 70597 Stuttgart, Germany; 3 Department of Oral and Maxillofacial Surgery, Dortmund Hospital and Faculty of Health, Witten/Herdecke University, Muensterstr. 240, 44145 Dortmund, Germany; 4 Department of Prosthodontics, Center for Dentistry and Oral Medicine (Carolinum), Goethe-University, Theodor-Stern-Kai 7, 60596 Frankfurt, Germany

**Keywords:** Maxillary sinus, Sinus lift, CBCT, Diagnostic imaging, Sinus septa

## Abstract

**Purpose:**

To analyze the visibility of the maxillary sinus septa (MSS) in panoramic radiography (PR) versus cone beam computed tomography (CBCT) and to investigate whether the buccal cortical bone thickness (BT) or the septa dimensions influence their visibility.

**Methods:**

Corresponding PR and CBCT images of 355 patients were selected and examined for MSS visibility. The septa dimensions (width, height, depth) and the BT were measured. Results were analysed statistically.

**Results:**

Comparing the corresponding regions on CBCT and PR, 170 MSS were identified; however, only 106 of these were also visible using PR. The MSS visibility was significantly higher on CBCT versus PR images (P1: p = 0.039, P2: p = 0.015, M1: p = 0.041, M2: p = 0.017, M3: p = 0.000), except region C (p = 0.625). Regarding the measurements of MSS dimensions, only the height in region M1 (p = 0.013) and the width in region P2 (p = 0.034) were significantly more visible on CBCT. The BT in the area of the MSS was found to have a marginal influence on its visibility on the PR images only in regions M3 and M1 (M3: p = 0.043, M1: p = 0.047). In terms of MSS visibility based on the dimensions, significance was found for all three influencing variables only in region P2 (width; p = 0.041, height; p = 0.001, depth; p = 0.007). There were only isolated cases of further significance: M3 for width (p = 0.043), M2 for height (p = 0.024), and P1 for depth (p = 0.034), no further significance was noted.

**Conclusion:**

MSS visibility appears significantly higher on CBCT versus PR images. It is concluded that the septa dimensions and BT can influence MSS visibility on PR images just in certain regions.

**Graphical Abstract:**

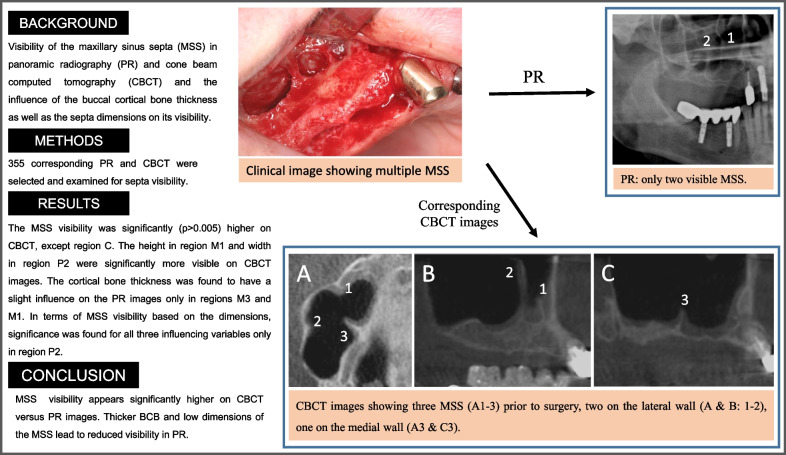

## Introduction

A common limitation when inserting dental implants is an insufficient bone supply in the edentulous area [1]. To restore lost teeth through implant-supported dentures in the posterior region of the upper jaw, sinus floor elevation is a common technique used in dental surgery to achieve an elevation of the maxillary sinus [2–4]. While the method is predictable and has high success rates, various complications have been documented either during surgery or the post-operative period [5].

**Fig. 1:**
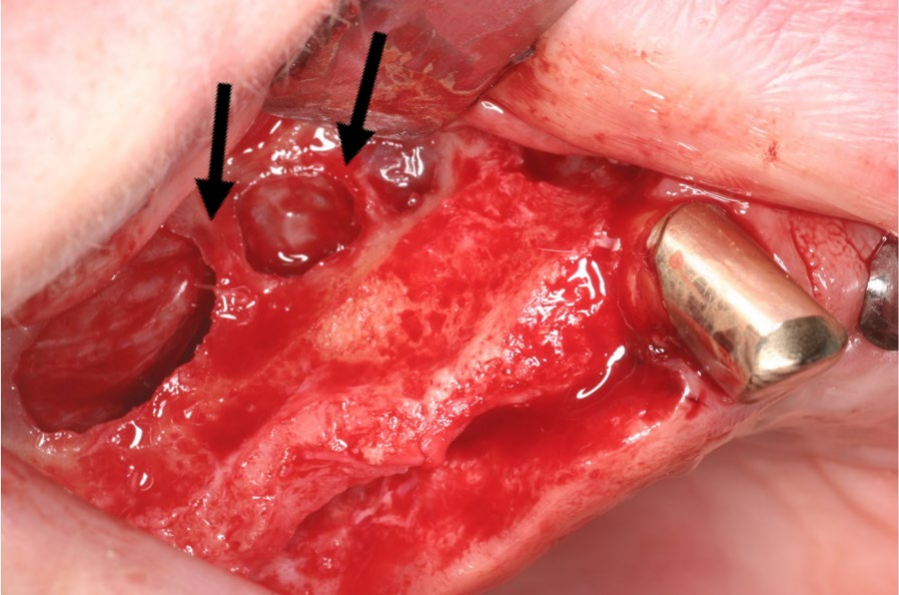
Clinical image showing multiple septa of the right maxillary sinus

The antral septum, a commonly found anatomical variation, has been related to the occurrence of membrane perforation during sinus augmentation [6]. Figure 1 shows the clinical situation during sinus lift surgery that was done with several prepared septa and a complex spatial configuration of the maxillary sinus. Complications arising from sinus floor elevation can be avoided by means of maxillary sinus examination in the pre-operative period [7].

The literature recommends that a thorough radiological examination of the maxillary sinus be conducted before the surgical procedure for sinus floor elevation to prevent possible complications [8, 9].

Both panoramic radiography (PR) and/or cone-beam computed tomography (CBCT) are often used and are advantageous tools for a successful diagnosis in oral and maxillofacial surgery and for general pre-operative planning [10, 11]. However, due to the tomographic nature of the technique, on PR images, only structures located within the tomographic plane are clearly delineated [12]. Many studies have identified the comparative superiority of CBCT over PR for detecting anatomical structures and for planning the insertion of dental implants in the maxilla [13–15].

Previous studies mentioned above emphasize the importance of CBCT diagnostics before a sinus lift surgery, but comparative studies directly comparing the detection accuracy of MSS between CBCT and corresponding PR images have still not been conducted. Likewise, the reason for the better visibility of septa in CBCT has not been investigated.

The present study presents an analysis of whether the visibility of the maxillary sinus septa (MSS) is comparable when using either panoramic radiography (PR) or cone beam computed tomography (CBCT) and also investigates whether the visibility on PR and CBCT is dependent on cortical bone thickness in the maxilla or the actual septa dimensions.

The hypothesis stated that MSS would be more commonly identified on CBCT versus PR images and that the visibility of the septa is influenced by the thickness of the buccal cortical bone in the maxilla and the size of the septa.

## Methods

Ethics approval was granted by the Committee of the Baden–Württemberg Medical Association (F-2014-006-z). The study was conducted in accordance with the ethical standards of the 1964 Declaration of Helsinki [16]. The present analysis was designed as a retrospective study. Between 2010 and 2017, patients who underwent both PR and CBCT scans were selected from the database of a private practice in Stuttgart.

The PR images used in this study were obtained by means of the Orthophos D 3297 X-ray unit (Sirona dental Systems GmbH, Bensheim, Germany) and saved on an imaging plate (Vistascan View, Dürr Dental, Bietigheim-Bissingen, Germany). The exposure parameters were established at a tube voltage of 60 kV, a current of 10 mA, and an exposure time of 16.4 s. These were read out with an imaging plate scanner (Vistascan Combi Plus, Dürr Dental). The evaluation was conducted using radiographic software DBSWin (version 5.1.1; Dürr Dental SE, Bietigheim-Bissingen, Germany).

The CBCT images were recorded using a Gendex CBX-500™ (KaVo Dental GmbH, Biberach, Germany). The acquisition parameters consisted of a tube voltage of 90 kV and an exposure time of 8.9 s with a 0.3 mm resolution using a field of view (FOV) of 6 or 14 cm (diameter) and 5–8.5 cm (height). The images were evaluated with the i-cat Viewer software (Imaging Sciences International, Hatfield, PA, USA).

The substantiating indications for the radiographs were obtained independently of the study. All images were taken by an expert in dental radiography. The examiner had the necessary qualifications and competence for CBCT and was briefed by an expert in the field of dental radiography and CBCT prior to the beginning of the study. To verify the reliability of the radiographic measurements and evaluations, multiple assessments were performed on 20 randomly selected patients. A reliability analysis (Cohen’s kappa coefficient) was conducted in a darkened room (< 1000 lx) using an accredited diagnostic monitor (EIZO FlexScan S2000 1024 × 1280 pixels) according to the radiographic instructions and under standardized conditions. Measurements were taken over a maximum of 6 h per day, including a 30-min break every 2 h. Inter-rater reliability of the outcomes between the examiner and expert was established. Furthermore, all images were examined a second time by the same examiner following an interval of two weeks for the calculation of intra-rater reliability. For both intra- and inter-observer reliabilities, kappa coefficients were computed.

**Fig. 2:**
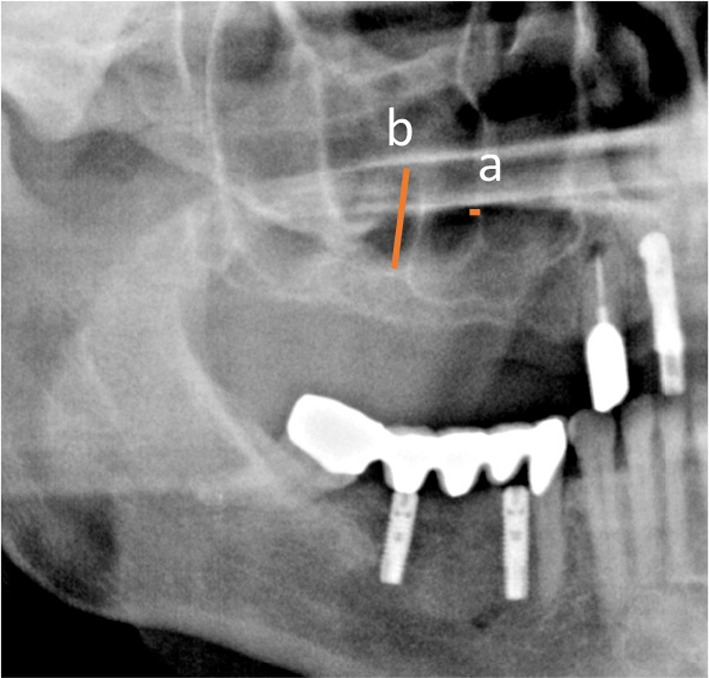
Panoramic radiographic (PR) image of the right maxillary sinus with measurements of the width (**a**) and height (**b**) of the septa. On the PR image, only two septa are clearly visible

**Fig. 3:**
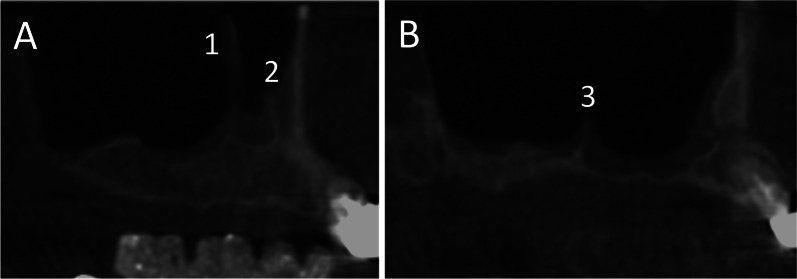
CBCT images of the right maxillary sinus from the same patient. Three septa are visible in the sagittal slices, two on the lateral wall (A1, A2) and one on the medial wall (B3) of the maxillary sinus

**Fig. 4:**
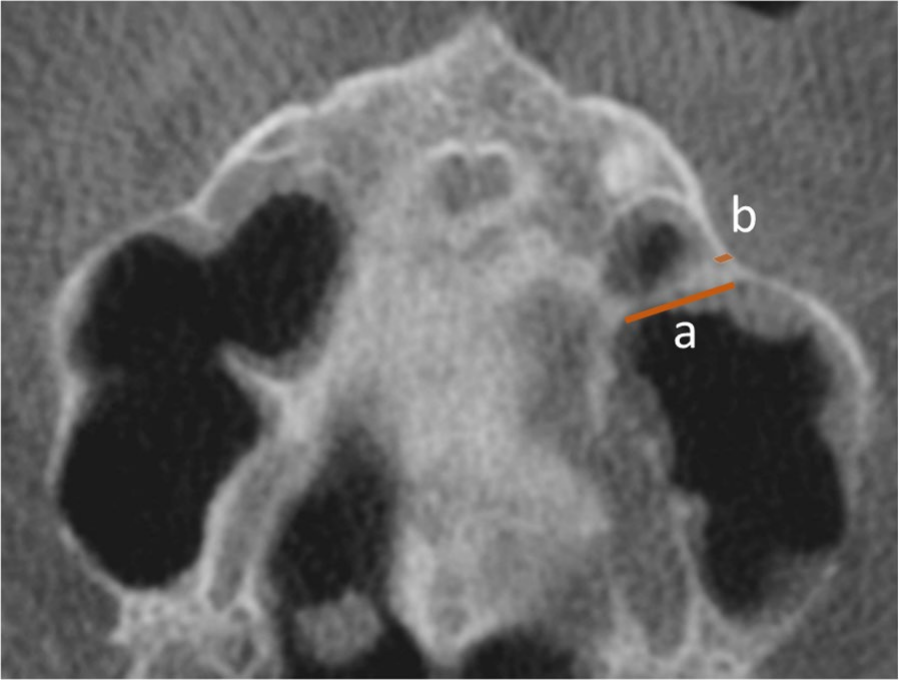
Cone-beam computed tomography (CBCT) image of the maxillary measuring the septum in the oro-vestibular extension (**a**) and the thickness of the buccal cortical bone (**b**): coronal slice

Patient data were anonymized, and the radiographic images were numbered. A chart was used to process the patient data and the radiographic indications using Microsoft Excel 2016 (Microsoft Inc., Redmond, Washington, USA). Both the PR and CBCT images (Figs. 2 and 3, respectively) were evaluated with respect to the detectable presence of the MSS on both the left and right sides. For diagnostic purposes, the sinuses were divided into the following sections: C canine, P1 and P2 first and second premolars, respectively; M1 first molar, M2 second molar; and M3 third molar. Thus, up to six different regions were assessed per jaw and per exposure. Sections that were not visible on both images were excluded from the evaluation. In addition, the width and height of the septa were measured on PR and CBCT images (Figs. 2 and 3, respectively). Additionally, on CBCT images, the buccal–oral extent (depth) of the MSS (Fig. 4a) was determined in addition to the thickness of the buccal cortical bone in the region of any septa (Fig. 4b).

The first step was to establish a diagnosis based on the PR images. This step was followed by a diagnostic analysis of the corresponding regions on the CBCT images. The findings were fed into a mask that had been specially developed by the Institute for Statistics (MediStat GmbH, Kornshagen, Germany) before being analysed by MediStat GmbH (Kronshagen, Germany) using the software SPSS Statistics 25 (IBM Corporation, Armonk, New York, USA).

The CBCT and PR measurements were tested using the Wilcoxon test for pair differences for variations. Two independent samples were compared by means of the Mann–Whitney U-test. To examine correlations between quantitative, not normally distributed parameters, a rank correlation analysis was carried according to Spearman.

The McNemar and the chi-squared tests were applied for statistical testing of the working hypothesis (differences between imaging techniques regarding the visibility of the sinus septa). A p-value of ≤ 0.05 indicated the presence of a significant difference.

Results with explorative and descriptive characteristics were expressed as absolute values (quantitatively with the mean and standard deviation) and incidence values (percentage).

## Results

For inclusion in the present study, 549 patients were initially selected from the database of a dental practice in Stuttgart, Germany after patients underwent PR and CBCT imaging between February 2010 and January 2017. Both PR and CBCT images were already available prior to the commencement of the study. Three-hundred thirty-seven patients met the inclusion criteria, of whom 173 patients were female and 165 were male patients. The average age of female patients was 62.1 years, and the average age of male patients was 58.5 years. For both intra- and inter-observer reliabilities, Cohen’s kappa coefficients were computed as 1.0 with a 95% confidence interval of 0.92–1.00.

**Fig. 5:**
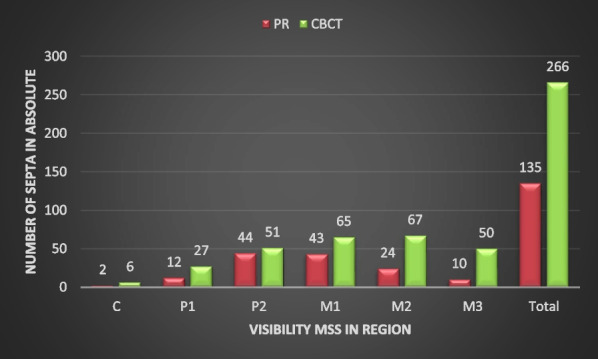
Visibility of the maxillary sinus septa (MSS) panoramic radiography and cone-beam computed tomography (PR and CBCT, respectively) absolute values. C canine, P1 and P2 first and second premolar, M1 first molar, M2 second molar, and M3 third molar

In the study, septa were found in all examined regions, both on PR and in CBCT images. Figure 5 shows the frequency of detected MSS in PR (n = 135) and CBCT (n = 266) in absolute numbers. It turns out that almost twice as many septa were detected in CBCT than in PR images. It is striking that most of the septa in PR images were found in regions P2 and M1, while in CBCT images this was the case in regions M1 and M2. If one puts the number of patients included in the study (n = 337) in relation to the septa found in the CBCT (n = 266), septa were found in 78.93% of the cases. However, since septa were found in several regions in one patient, it made more sense to use the number of regions as a basis. Based on the regions (n = 2022), septa were found in 13.15% of regions. Thirteen of the detected MSS on PR images were not visible on CBCT images (one in region M3, five in M2, six in M1, and one in C).

The mapping of jaw sections on the CBCT and PR images was audited prior to the definitive evaluation. For many patients, the field of view (FOV) on the CBCT image was smaller than on the PR, so it was not possible to account for all sections in these patients.

Tables 1 and 2 show the visibility of the MSS on the PR and in corresponding regions on the CBCT images, respectively. On PR images (Table 1), the MSS was detected in 106 sections of the maxilla (21.5%). Table 3 shows the visibility of MSS on the CBCT images. In this study, 170 MSS (34.5%) were detected (Table 2).

**Table 1:** Visibility of the MSS in PR in percent of the total

	Quantity	% of the total	% valid cases
PR: MSS visible			
No	234	47.5	68.8
Yes	106	21.5	31.2
Total	340	69.0	100.0
System error	153	31.0	
Total	493	100.0

**Table 2:** Visibility of the MSS on CBCT images in percent of the total

	Quantity	% of the total	% valid cases
CBCT: MSS visible			
No	170	34.5	48.3
Yes	170	34.5	48.3
Non-evaluable	12	2.4	3.4
Total	352	71.4	100.0
System error	141	28.6	
Total	493	100.0

**Table 3:** Thickness of the buccal cortical bone in mm

	P1	P2	M2
CBCT = PR	2.35 (SD ± 1.09)	1.96 (SD ± 0.67)	1.60 (SD ± 0.5)
CBCT ≠ PR	2.19 (SD ± 0.61)	1.95 (SD ± 0.53)	1.63 (SD ± 0.41)

In all regions, a high significance of the visibility of MSS in favor of CBCT, with the exception of the canine region. The values calculated using the McNemar test are listed: (1) C: p = 0.625, (2) P1: p = 0.039, (3) P2: p = 0.015, (4) M1: p = 0.041, (5) M2: p = 0.017, and (6) M3: p = 0.000.

### Influence of the thickness of the cortical bone on the visibility of the MSS

**Fig. 6:**
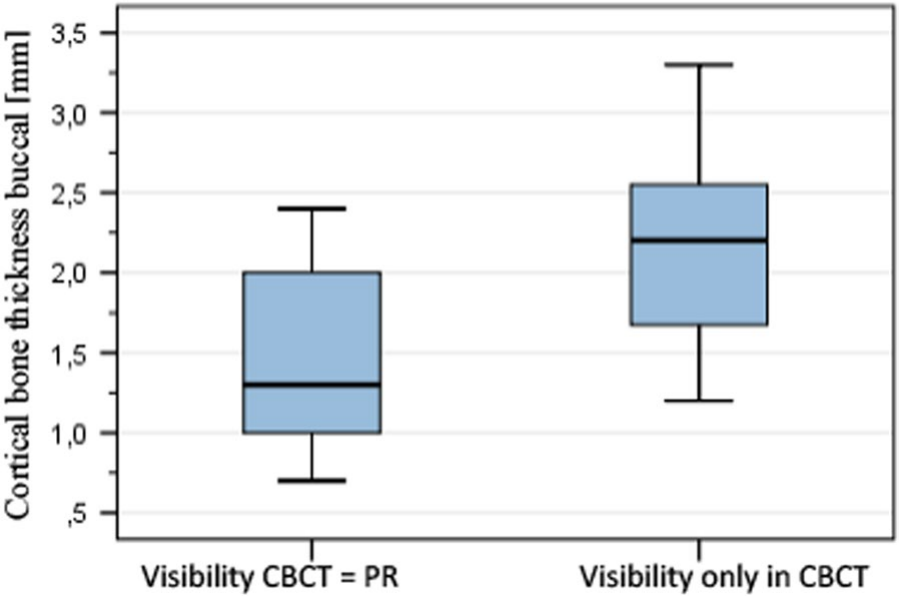
Influence of the buccal cortical bone thickness on the visibility of MSS in CBCT and PR in region M3

In region M3, in which the MSS is detected considerably less frequently in the PR (Fig. 5), the thickness of the buccal cortical bone in such cases in which visibility in the CBCT and PR are equal was 1.45 mm ± 0.662 (mean ± standard deviation [SD]). In the group with divergent visibility, the compact thickness was 2.15 mm ± 0.571) as shown in Fig. 6. The buccal cortical bone thickness shown in region M3 produced a significant influence on the consistency of MSS visibility in CBCT and PR measurements (Mann–Whitney U test, M3: p = 0.043). The same result was found in terms of the visibility of the MSS in region M1 in which the difference between PR and CBCT was not quite as large as in region M3. In this situation, the thickness of the buccal cortical bone influences the visibility of the MSS significantly (Mann–Whitney U test, M1: p = 0.047). In the case of equal visibility in PR and CBCT, the thickness was 1.56 mm ± 0.37 mm and if MSS were only visible in CBCT, it was 2.1 ± 0.86 mm.

Thus, the visibility of the MSS in PR in both regions (M1 and M3) was significantly lower the thicker the cortical thickness was.

For other regions, namely P1, P2, and M2 (Table 3), the differences were very small, and no significant differences could be found (Man–Whitney U test; P > 0.05). No further evaluations were carried out for the canine region because the number of visible septa in CBCT was very small and not relevant.

### Influence of depth, height, and width on the visibility of the MSS

Table 4 depicts the mean depth value of the MSS. The highest depth was found in region M2 with 8.07 ± 3.27 mm and the lowest in region C with 5.62 ± 3.53 mm. In the other regions, the depth was found about 8 mm. Table 4 also shows the distribution of measurements in percentiles. The 25th percentile showed depths between 2.54 mm in region C that increased to an M3 of 5.87 mm. In the 50th percentile, the numbers were inhomogeneous. In the 75th percentile, the depths were more homogeneous and moved around 10 mm.

**Table 4:** The mean depth and standard deviation in addition to maximum and minimum of the septa in CBCT (expressed in mm)

Region	N	Mean value	SD	Minimum	Maximum	Percentile		
						25	50	75
C	6	5.62	3.53	1.34	10.82	2.54	4.90	10.21
P1	27	7.97	4.44	1.00	25.20	4.92	7.45	9.50
P2	51	7.69	4.42	0,60	17.73	4.82	4.14	9.72
M1	65	7.66	3.75	0.85	20.00	5.73	7.25	9.39
M2	67	8.07	3.27	1.00	18.72	5.81	8.10	9.75
M3	50	7.29	2.78	0.60	13.57	5.87	7.20	9.30

**Fig. 7:**
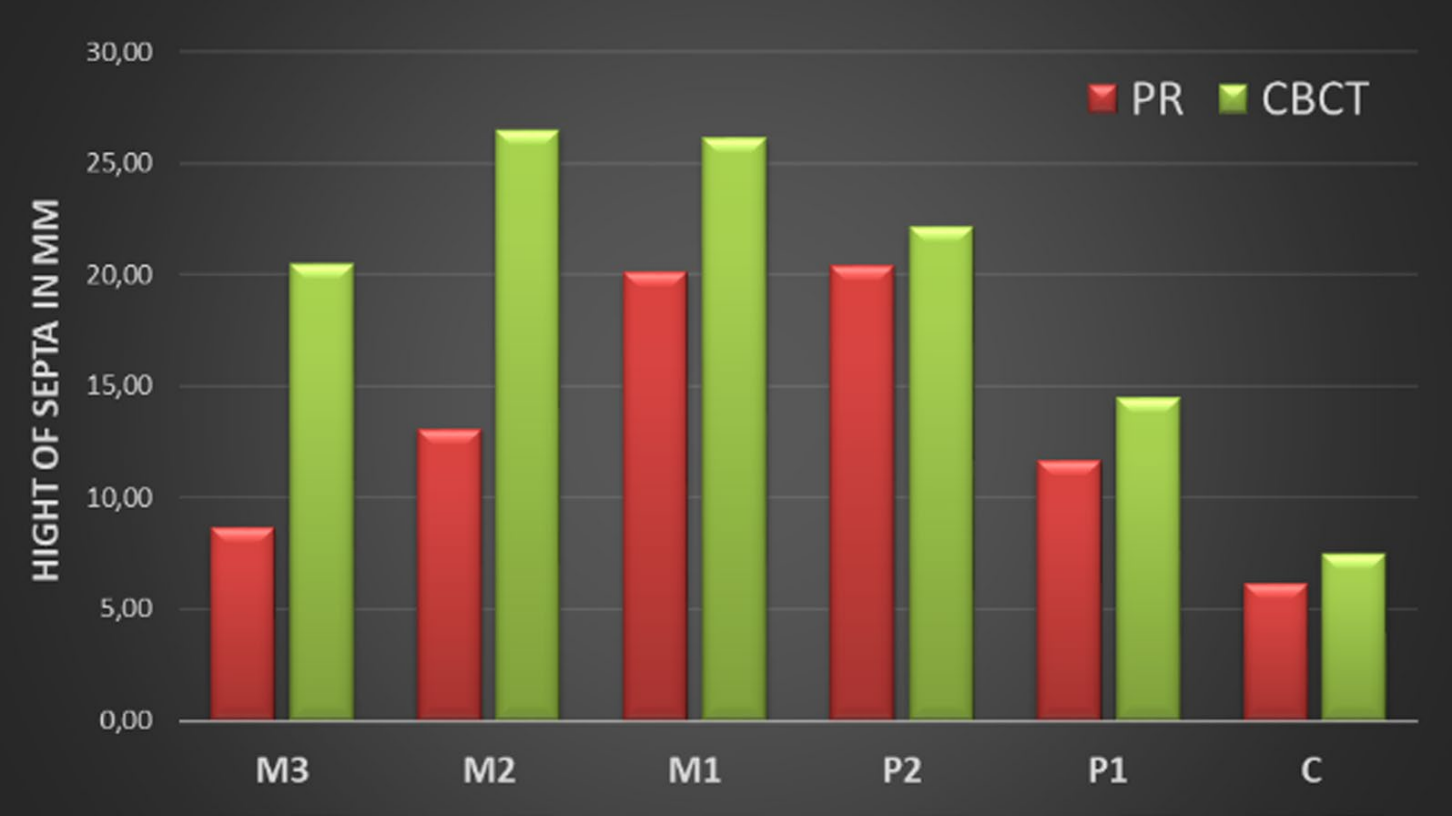
The height of the septa on PR versus CBCT images

**Fig. 8:**
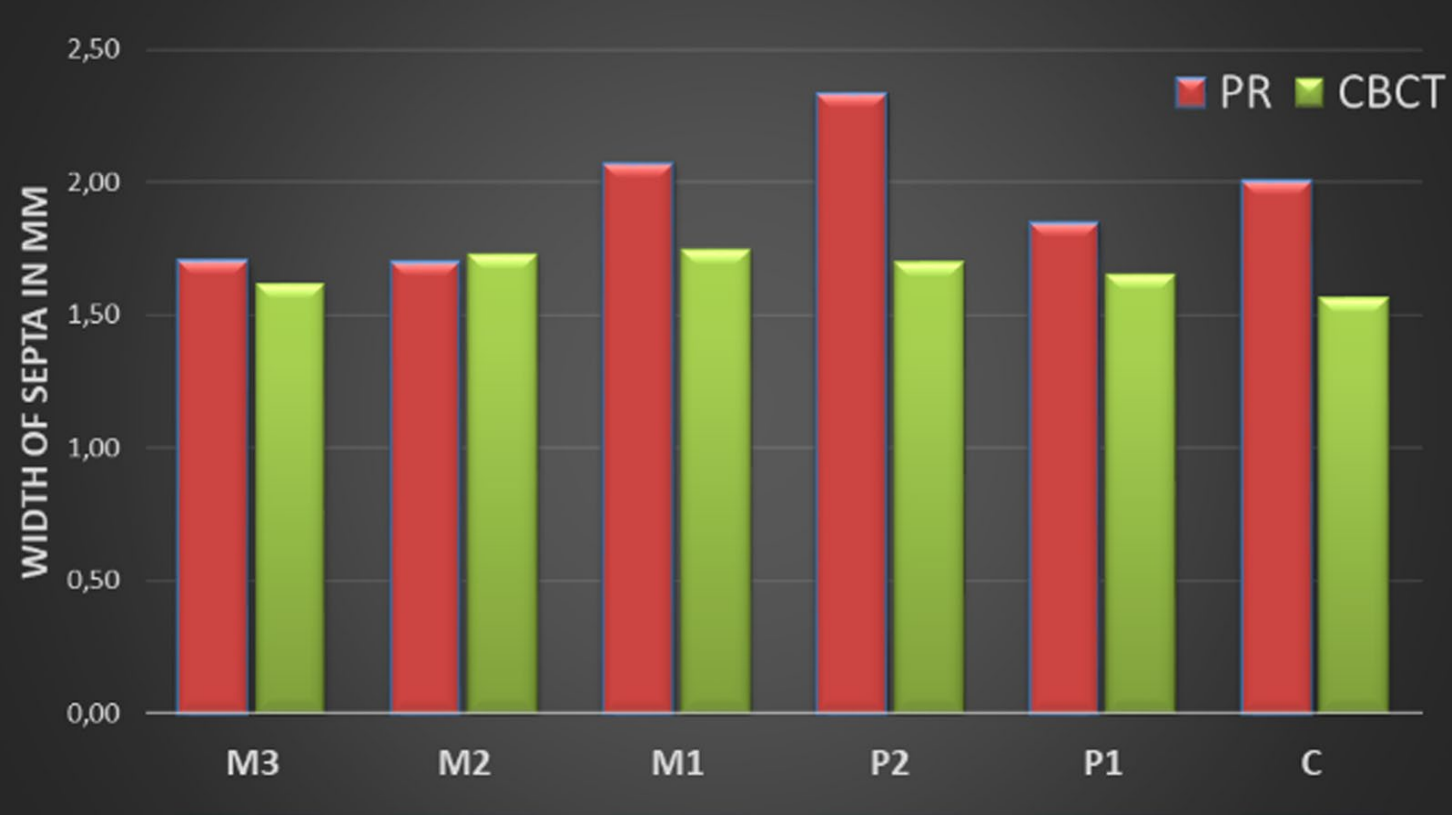
The width of the septa on PR versus CBCT images

Figure 7 and 8 provide detailed information about the width and height of the septa in the examined regions on the PR and CBCT images. At first glance, it is noticeable that the height of the septa in the PR images was measured lower in all regions than in the CBCT images (Fig. 7), while the values of the measured widths differ only slightly (Fig. 8). Although the height differences in the septa were very clear in some cases, statistical significance was only found in region M1 (Wilcoxon test: p = 0.013). Regarding the measured widths, statistical significance was only found in the P2 region (Wilcoxon test: p = 0.034).

**Fig. 9:**
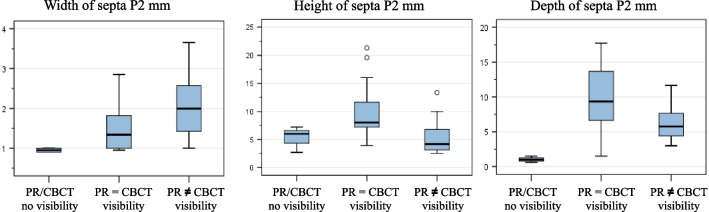
Influence of width, height, and depth on the visibility of the septa on PR and CBCT images (in mm)

Figure 9 shows the influence of width, height, and depth on the visibility of the septum between PR and CBCT images in region P2. Only in this region, was there significance for all three influencing variables (Mann–Whitney U test; width p = 0.041, height; p = 0.001, and depth; p = 0.007). There were only isolated cases of further significance in region M3 for width (Mann–Whitney U test; p = 0.043), in region M2 for height (Mann–Whitney U test; p = 0.024), and in region P1 for depth (Mann–Whitney U test; p = 0.034); otherwise, no further significance was noted.

## Discussion

The aim of the present comparative study was to analyse whether the visibility of the MSS is comparable using PR versus CBCT and also to investigate whether the visibility on PR and CBCT images depends on cortical bone thickness in the maxilla or the actual dimensions of the septa. The hypothesis stated that the MSS would be more commonly identified on CBCT versus PR scans and in addition the visibility of the septa is influenced by the thickness of the buccal cortical bone wall of the maxilla and the dimension of the septa.

For the present study, 549 patients were initially selected from the database of a dental practice in Stuttgart, Germany after they underwent PR and CBCT imaging between February 2010 and January 2017. Both PR and CBCT images were already available prior to the start of the study. Three-hundred thirty-seven patients fulfilled the inclusion criteria of whom 173 patients were female and 165 were male patients. The average age of female patients was 62.1 years, and the average age of male patients was 58.5 years.

In the preliminary stages, measurement integrity was verified by an expert. The measurements were repeated at 2-week intervals. For both intra- and inter-observer reliability, Cohen’s kappa coefficient was computed as 1.0 with a 95% confidence interval of 0.92–1.00. Thus, a high degree of agreement was obtained.

The findings of our study indicate that on the CBCT images, the MSS could be detected in 266 cases in the maxillary sinus. However, only about half of these (135) were also visible on PR images (Fig. 5). Thirteen of the detected MSS on PR images were not visible on the CBCT images. A direct comparison of the corresponding images revealed that this difference may be caused by translucencies, such as those due to the hard palate or by imaging the dorsum of the tongue. Both the palate and dorsum are superimposed on the maxillary sinus on PR images and could result in misdiagnoses.

Sinus lifting has become a common surgical intervention aimed at producing an increase in alveolar bone height prior to dental implant placement in the posterior maxilla [17]. However, the occurrence of specific intraoperative complications, such as Schneiderian membrane perforation, bleeding from the antral alveolar artery, and/or the presence of septa must be considered [7, 18, 19]. Septa are common anatomical structures and are most often found in the first or second molar region on the floor of the maxillary sinus [15]. By contrast, Ulm et al. [19] and Pommer et al. [20] report that septa can occur in all maxillary sinus regions. Systematic reviews reported a frequency of the occurrence of maxillary sinus septa between approximately 13%–35% [20, 21]. The group around Irinakis et al. even report a 48.1% occurrence of septa [22].

Wen et al. performed a literature search and found that sinus augmentations are subject to an increased complication rate in the presence of septa [6]. The orientation of the septa can cause sinus augmentations to be even more difficult [6, 22].

Based on the results of their studies, some authors recommend a classification system of septa and treatment strategies to reduce complications during sinus augmentation [6, 21, 22].

A thorough knowledge of the anatomy of the maxillary sinus and its possible variations is essential to prevent complications during sinus augmentation [23].

Both PR and CBCT are available for diagnosis and planning before dental procedures [10, 11]. Due to the size and distribution of anatomical structures, not every area of interest can be clearly visualized on PR images and can therefore be negatively influenced by various structures [24]. In their experimental and comparative diagnostic study, Dau et al. found that PR alone remained insufficient for evaluating pathologies in the maxillary sinus. Furthermore, the presence of pathologies can affect the visibility of anatomical structures [25]. Pommer et al. concluded. That septa diagnosis using panoramic radiographs yielded incorrect results in 29% of cases [20]. Anamali et al. report in their study that CBCT images show highly beneficial information regardless of the presence of intra-sinus pathology [26]. CBCT images appear to enable an examiner-independent assessment of anatomical structures as they leave little room for interpretation of the findings [27]. Therefore, some authors suggest a thorough three-dimensional radiographic examination of the sinus prior to surgery [15, 20, 28, 29].

The results of our study show that septa generally occur in all regions of the maxillary sinus, both in CBCT and in PR. In their systematic reviews, Pommer et al. [20] and Maestre-Ferrín et al. [21] provide similar results. In our study, septa were found in 13.15% of the cases on CBCT images based on the regions (n = 2022). On CBCT images, however, the septa were most frequently visible in regions M2 and M1 followed by P2 and M3 at a similar level. The septa were observed much less frequently in region P1 and only to a negligible degree in region C1. The study thus shows similar observations regarding the visibility of the MSS and is confirmed by the results of the above-mentioned studies [19–21]. The visibility of the septa in PR images was highest in the P2 region. Otherwise, the septa behave similarly on CBCT images (Fig. 5). Overall, the number of septa observed on CBCT images was approximately twice as high as on PR images (Fig. 5). When directly comparing the visibility of septa between the corresponding regions, septa were detected in 34.5% of cases on CBCT and in 24.5% of cases on PR images (Tables 1 and 2, respectively). In our study, CBCT images show a significantly higher detectability of MSS in all regions (p < 0.05) compared to PR images with the exception of the canine region (p > 0.05).

For daily practice, the results of our study can be interpreted as follows: the differences in the detectability of MSS between PR and CBCT in the anterior region are smaller than in the posterior region. While in regions M3 (p < 0.000) and M2 (p < 0.017) the detectability of MSS in CBCT is significantly higher than in PR, this difference is not significant in region C (p > 0.625). In regions M1 (p < 0.041) and P1 (p < 0.039) the significance is marginal. Accordingly, the importance of the PR for detecting the septa in the anterior region is considered to be high. In the posterior area, CBCT would be more useful to identify the septa.

Regarding the influence of the thickness of the buccal cortical bone on the visibility of the MSS in a direct comparison between CBCT and corresponding PR images, there is a lack of specialist literature. In our study, the buccal cortical bone thickness showed in region M3 (p = 0.043) and M1 (p = 0.047) a significant influence on the consistency of MSS visibility in CBCT and PR measurements, if they were only visible in CBCT. The visibility of the MSS was significantly lower in PR the thicker the buccal cortical bone was. In all other regions no significant correlation was found between the visibility of the MSS and the buccal cortical bone thickness (p > 0.05).

The results here are a bit confusing. It could actually be the case that the thickness of the buccal compact influences the visibility of the septa only in regions M3 and M1. The study by Simsek et al. provides an indication of this [30]. The authors noted that the highest thickness of the lateral wall of the sinus was found in the first molar region. However, this does not explain the high significance in region M3.

On the other hand, it is also possible that the visibility of MSS in PR is influenced by anatomical variations as described by Shiki et al., which are stronger in the posterior region of the maxillary sinus [24]. The same rationale applies to the presence of pathologies that affect the visibility of anatomical structures [25–27]. These aspects were not examined in our study and thus cannot be sufficiently proven by studies. Further studies are necessary to investigate these aspects.

Another aspect of our study was to investigate the influence of the size of the septa on their visibility between the PR and CBCT images. The results of our study show that the measured values for the height of the septa on the CBCT (range 7.44–26.48 mm) images are higher than on PR (range 6.17–20.36 mm) images in all regions. However, significance could only be shown for the measurements in region M1 (Fig. 7; Wilcoxon test: p = 0.013). For the width of the septa, the values between PR (range 1.70–2.33 mm) and CBCT (range 1.57–1.74 mm) images differed only slightly. Significance was only found for region P2 (Wilcoxon test: p = 0.034) as shown in Fig. 8. Furthermore, the depth of the septa was considered a possible influencing factor for their visibility on PR images. The lowest values for the depth of the septa were measured in region P1, and the values were similar in all other regions.

The measured values for the dimension of the septa as an influencing factor for their visibility on PR images do not reveal a clear trend. Significance was found in all three measurements for the visibility of MSS in PR only in P2 (Mann–Whitney U test, width p = 0.041, height p = 0.001, and depth p = 0.007). Isolated cases of further significance in region M3 for width (Mann–Whitney U test p = 0.043), in region M2 for height (Mann–Whitney U test p = 0.024), and in region P1 for depth (Mann–Whitney U test p = 0.034) were found.

In the specialist literature, septa height showed great variability with a mean value of 8.72 mm (SD ± 4.26; range 3.7–18.4 mm) [23]. In a systematic review, Pommer et al. report that the height of the septa is on average 7.5 mm [20]. In their literature review, Maestre-Ferrín et al. provide values between 2 and 13 mm for the height of the septa [21]. The values measured in our study correspond to the previously reported studies. Nevertheless, no direct comparison was made between PR and CBCT in these studies.

The results of our study are consistent with the results of other studies in terms of the frequency and distribution of MSS [19–21]. Our study shows that septa generally occur in all regions of the maxillary sinus, basically being visible both on CBCT and PR images. However, septa can be detected almost twice as often on CBCT compared to PR. For the influence of the dimension of the septa on their visibility, no clear trend could be found in our study. Only in isolated cases was there a significant influence due to the septa dimension. This aspect should be investigated in more detail in further studies. The influence of the thickness of the buccal cortical bone on the visibility of MSS seems to be more promising. A significant influence due to the thickness was observed in regions M1 and M3. This aspect has not yet been investigated in the literature.

Operations on the maxillary sinus are complicated by the presence of antral septa, the visibility and dimensions of which determine the degree of surgical difficulty. For the purposes of reducing such complications, it is recommended that a thorough radiological assessment of the maxillary sinus be carried out in the sinus region prior to surgical intervention [8, 9]. The location, dimension and course of the septa can have an influence on the planning of maxillary sinus surgery. Wen et al. [6] were able to show in their literature review that knowledge of these parameters is essential when developing a strategy for planning maxillary sinus surgery.

In principle, it must be noted that CBCT is clearly superior to PR for the detection of MSS. Similar results were reported in the systematic review by Pommer et al. The authors found that the diagnosis of MSS by PR was incorrect in 29% of cases, which is a lot [20]. One reason for this is that, due to the technique, only structures that lie within the tomographic plane are clearly visible on PR images. Objects that are behind the tomographic plane appear wider and those in front of them appear narrower [12]. Horizontal and vertical distortions can be observed outside the tomographic plane. These biases are the reasons why the measurements in PR are unreliable. Incorrect positioning of the head can also lead to distortion of the anatomical structures. Incorrect positioning of the head relative to the midline causes horizontal errors, so that anatomical structures in the posterior region appear wider or narrower. Incorrect positioning of the head relative to the horizontal plane causes vertical errors, making structures appear longer or shorter [12]. It also plays a role that CBCT allows for fewer interpretations and the findings are less dependent on the examiner [27].

However, CBCT also has some disadvantages, e.g. CBCTs show poor soft tissue contrast [31] and artefacts, especially when metal or root canal fillings are in the area of interest [32]. Another typical problem with CBCT are artefacts that arise from movements of the head. The cause of this is insufficient fixation of the head. But the time required to produce a CBCT can also be a possible cause. Since larger FOVs take longer to produce, CBCTs with small FOVs focusing on the region of interest are more advantageous [33].

Finally, the question arises as to whether CBCT can have an influence on the planning of a surgical procedure on the maxillary sinus. The study by Kaeppler et al. [34] showed that creating a CBCT can have an influence on treatment planning. However, this only affected 9.5% of cases with suspected mandibular fractures. However, this aspect was not examined in our study. It remains to be seen whether further studies will provide clarity for other surgical indications.

Further studies are necessary here.

Therefore, our hypothesis that MSS would be more commonly identified on CBCT versus PR images can be verified. The assumption that the dimension in addition to the thickness of the buccal cortical bone influences the visibility of the septa can only be verified to a limited extent. Here, not all regions show significance.

## Conclusion

The present study is based on a direct comparison of corresponding PR and CBCT images and shows that CBCT is superior to PR in terms of the detectability of MSS. Septa are common in the maxillary sinus and are introduced as a complication factor during sinus augmentation. In terms of surgical planning prior to sinus augmentation, CBCT provides the necessary information to avoid complications early in the peri-operative planning phase.

In recent years, the use of CBCT has become increasingly practicable and popular. Given that this procedure involves increased ionising radiation in comparison to PR, CBCT should only be done in cases in which the potential patient benefits outweigh the risks. Nonetheless, ethical and radiobiological aspects must be considered in accordance with the ‘as low as diagnostically acceptable’ (ALADA) principle.

Based on the present data, the authors conclude that CBCT shows significantly more surgically relevant information in implant dentistry, in particular for maxillary sinus diagnosis and can be recommended for visualising the antrum septa when surgically planning sinus augmentation procedures.

## Data Availability

The original datasets analysed in the current study are available on reasonable request from Dr. Ali Reza Ketabi.

## Abbreviations

CBCT: Cone-beam computed tomography
PR: Panoramic radiography
MSS: Maxillary sinus septa
SL: Sinus lifting
sec: Second
kV: Kilo volt
mA: Milli ampere
mm: Millimetre
cm: Centimetre
FOV: Field of view
SD: Standard deviation
